# Developing a Low-Cost Passive Method for Long-Term Average Levels of Light-Absorbing Carbon Air Pollution in Polluted Indoor Environments

**DOI:** 10.3390/s20123417

**Published:** 2020-06-17

**Authors:** Lara P. Clark, V. Sreekanth, Bujin Bekbulat, Michael Baum, Songlin Yang, Pao Baylon, Timothy R. Gould, Timothy V. Larson, Edmund Y. W. Seto, Chris D. Space, Julian D. Marshall

**Affiliations:** 1 Department of Civil & Environmental Engineering, University of Washington, Seattle, WA 98195, USA; lpclark@uw.edu (L.P.C.); sree.hcu@gmail.com (V.S.); bujinb@uw.edu (B.B.); yangsonglin@tsinghua.org.cn (S.Y.); tgould@u.washington.edu (T.R.G.); tlarson@uw.edu (T.V.L.); cspace70@gmail.com (C.D.S.); 2 Center for Study of Science, Technology & Policy, Bengaluru 560094, India; 3 Axon Engineering LLC, Bellevue, WA 98006, USA; mebaum@axonengineering.com; 4 Astronaut Center of China, Beijing 100094, China; 5 Department of Atmospheric Sciences, University of Washington, Seattle, WA 98195, USA; lbaylon@uw.edu; 6 Department of Environmental & Occupational Health Sciences, University of Washington, Seattle, WA 98195, USA; eseto@uw.edu

**Keywords:** household air pollution, indoor air quality, low-cost measurements, time-integrated average, exposure assessment, community monitoring, passive sampling, black carbon, brown carbon, fine particulate matter

## Abstract

We propose a low-cost passive method for monitoring long-term average levels of light-absorbing carbon air pollution in polluted indoor environments. Building on prior work, the method here estimates the change in reflectance of a passively exposed surface through analysis of digital images. To determine reproducibility and limits of detection, we tested low-cost passive samplers with exposure to kerosene smoke in the laboratory and to environmental pollution in 20 indoor locations. Preliminary results suggest robust reproducibility (*r* = 0.99) and limits of detection appropriate for longer-term (~1–3 months) monitoring in households that use solid fuels. The results here suggest high precision; further testing involving “gold standard” measurements is needed to investigate accuracy.

## 1. Introduction

Exposure to indoor air pollution causes 2–3 million premature deaths per year [1] and disproportionately impacts low-income households that rely on solid fuels in lower-income countries [2]. Light-absorbing carbon (LAC) air pollution includes carbon components of fine particulate matter (PM_2.5_), such as black carbon (BC) and brown carbon (BrC), that strongly absorb visible light [3]. LAC, which is a product of incomplete combustion, is a common air pollutant in households that rely on solid fuels and contributes substantially to climate-forcing [4] and public health risks [5]. 

Broad monitoring of long-term LAC is needed for assessing exposures [6], quantifying emissions [2,4], identifying local pollution sources [7,8], and evaluating impacts of interventions [9]. However, monitoring can be expensive (e.g., monitor costs: hundreds to thousands of United States Dollars [USD]) and logistically challenging (e.g., requiring power, laboratory facilities, and frequent sampling and maintenance) [10,11,12], particularly in resource-constrained and remote communities that rely on solid fuels [13], where indoor air pollution concentrations often far exceed health-based guidelines [14]. Developing low-cost and easy-to-use methods for longer-term LAC could enable broad deployment of household air pollution monitoring [15,16]. Specifically, combining passive sample collection with digital image-based analysis [17] could provide a low-cost and easy-to-use method for broadly monitoring long-term average LAC air pollution levels. 

Passive sample collection can provide a low-cost alternative to active sample collection for broadly monitoring long-term average air pollution [18,19,20]. Available methods for monitoring LAC (e.g., thermal-optical methods (e.g., OC-EC aerosol analyzer), photo-acoustic methods (e.g., photo-acoustic soot spectrometer), real-time optical methods (e.g., aethalometer), and filter-based time-integrated average optical methods (e.g., smoke stain reflectometer; optical transmissometer)) use active sample collection (i.e., a pump to draw an air sample through a PTFE or quartz filter or through filter tape) and generally provide shorter-term (~hours) measurements [21,22]. In contrast to active sample collection, passive sample collection (i.e., collection of PM through gravitational settling, inertia, and diffusion) does not require power, filters or other consumables, does not generate noise or heat, does not require frequent maintenance (e.g., for battery or filter replacement), and, in some cases, can provide longer-term (~days to months) measurements [16]. For monitoring PM and its components, passive samples have been collected using environmental (i.e., non-engineered or incidental) surfaces (such as walls [23] and birds’ feathers [24]) and using specialized (i.e., engineered) samplers (such as polycarbonate filter samplers for scanning electron microscopy (SEM) analysis [25,26], polyurethane foam (PUF) disk samplers [27], and PTFE or quartz filter samplers [28,29]). Based on evaluation with active samplers, passive samplers can reliably estimate long-term average PM concentrations [19,27].

Digital image-based analysis methods (i.e., analyzing images with computer software to quantify the reflectance or color of a sample) can offer a non-destructive alternative to thermal-optical analysis methods for LAC air pollution and a lower-cost alternative to established optical analysis methods for LAC air pollution [10]. Like other time-integrated average optical methods for monitoring LAC (e.g., smoke stain reflectometer), digital image-based methods estimate the reflectance or color of a sample collected on a filter (e.g., quartz or PTFE filter) and then estimate the LAC loading of the filter based on field calibration with other optical or thermal-optical methods for LAC. Digital image-based methods have been developed using cellphone cameras [10,30], digital cameras [22,31], colorimeters [32,33], and scanners [34,35,36] and have been tested in households that use solid fuels in China and India [22,36,37]. Based on evaluation with thermal-optical methods and time-integrated average optical methods for LAC, digital image-based analysis methods can reliably estimate shorter-term (~hours) average LAC concentrations [10,13,22,30,31,32,33,34,35,36].

Our objective was to develop a low-cost, easy-to-use method for long-term time-integrated average indoor LAC levels. We build on prior work by combining passive sampling with digital image analysis [17,24]: two low-cost methods which have been applied separately (but not together) for measuring LAC (or other components of PM) indoors. Previous studies have applied digital image analysis with active sample collection to measure LAC concentration based on filter reflectance [10,13,22,30,31,32,33,34,35,36], and, separately, previous studies have applied passive sample collection with other analysis methods (e.g., electron microscopy [19], chemical analysis methods [29]) to measure PM components. 

Our approach combined two low-cost methods (passive sample collection; digital image-based analysis [17,24]) for measurement of long-term average indoor LAC levels based on changes in reflectance of a passively exposed surface. We designed a specialized 3D-printed passive sampler (with materials that cost less than ~10 USD per sampler), and we tested samplers in the laboratory and the field to determine the reproducibility and limits of detection for measurement of reflectance. Here, we present the low-cost passive method, describe its reproducibility and its limits of detection (but not its accuracy), and discuss its potential suitability for long-term monitoring in polluted indoor environments.

## 2. Materials and Methods

### 2.1. Designing the Sampler 

#### 2.1.1. Concept and Design Criteria

The concept for the specialized passive sampler consists of a white exposure surface contained in protective casing. The sampler is placed indoors for several weeks to months, and, during this period, the white exposure surface darkens (i.e., decreases in reflectance) as particles are collected on the downward-facing exposure surface, generally via diffusion, in proportion to exposure to indoor LAC air pollution concentrations. To estimate the change in reflectance over time, the sampler is imaged with a digital camera in a lightbox, and images are analyzed using computer software. 

We developed the following design criteria for the specialized passive low-cost sampler for large-scale, long-term deployment indoors in resource-constrained and remote communities that rely on solid fuels:

1. Materials cost less than 10 USD per sampler.
2. Sampler parts (except for exposure surfaces and identifying labels) are re-usable and recyclable.
3. Samplers are fully passive during monitoring.
4. Samplers can be left in place for weeks to months indoors.
5. Samplers are easy to assemble and to deploy.

#### 2.1.2. Iterative Design Phases

**Figure A1:**
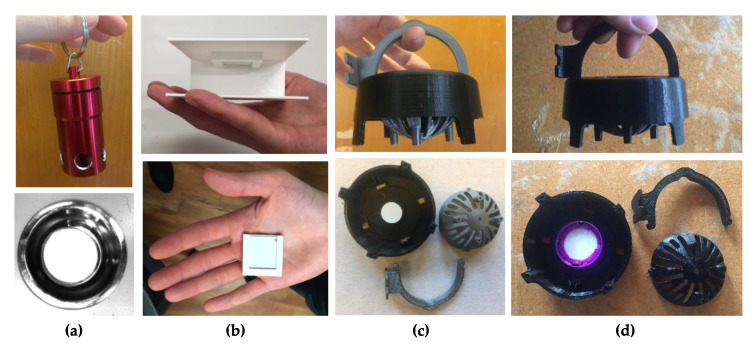
Early prototypes of the low-cost passive sampler, including: (**a**) samplers constructed by modifying existing low-cost products, such a pill bottle with drilled holes and a painted white stainless steel sampling surface (top) and an inverted steel bowl with quartz filter (bottom); (**b**) samplers with a 3D-printed rectangular casing and a painted white stainless steel sampling surface; (**c**) samplers with a 3D-printed circular casing and a paper filter exposure surface, protective cage, and hook for hanging; and, (**d**) samplers with a 3D-printed circular casing as in (**c**) with an added removable clamp for the paper filter exposure surface.

We prototyped and tested iterative designs for a low-cost passive sampler (Figure A1). Initial prototypes were constructed from existing low-cost commercial products or re-used packaging materials (e.g., metal bowls, plastic and metal pill bottles, glass jars). Later prototypes were specialized designs constructed with a 3D-printer. In early design phases, we tested multiple materials for the exposure surface (paper, stainless steel (coated with white paint), glass, and quartz) in a kerosene lamp smoke exposure chamber (details in Section 2.2.1) and in a diesel engine exhaust exposure chamber [38]. Similarly, we tested several approaches for protecting the exposure surface (e.g., from turbulence, from contact during handling, and from insects), including windshields, wire mesh cages, and 3D-printed plastic cages. 

In parallel with the iterative design phases for the low-cost passive sampler, we prototyped and tested iterative approaches for imaging the samplers with a digital camera and for analyzing images to estimate changes in sampler exposure surface reflectance. For stable and even lighting conditions in the digital images, we designed and tested lightboxes constructed from low-cost materials [17,22,24]. Other aspects of the approach we tested include number of photographs analyzed (i.e., multiple frames from the same time-point, range: 1 to 1000 frames per time-point), area of sampler exposure surface analyzed, camera model (e.g., cellphone cameras (iPhone 6, Apple Inc., Cupertino, CA, USA; Galaxy Prime, Samsung Electronics, Suwon, Korea), color digital cameras (Nikon D7100, Nikon Corporation, Tokyo, Japan), grayscale digital cameras (Basler acA3800-14um and Basler puA2500-14um, Basler AG, Ahrensburg, Germany)), camera settings (e.g., exposure time), and reference approach for images (e.g., using a reference grayscale card and/or an unexposed reference sampler).

#### 2.1.3. Design of the Sampler

**Figure 1:**
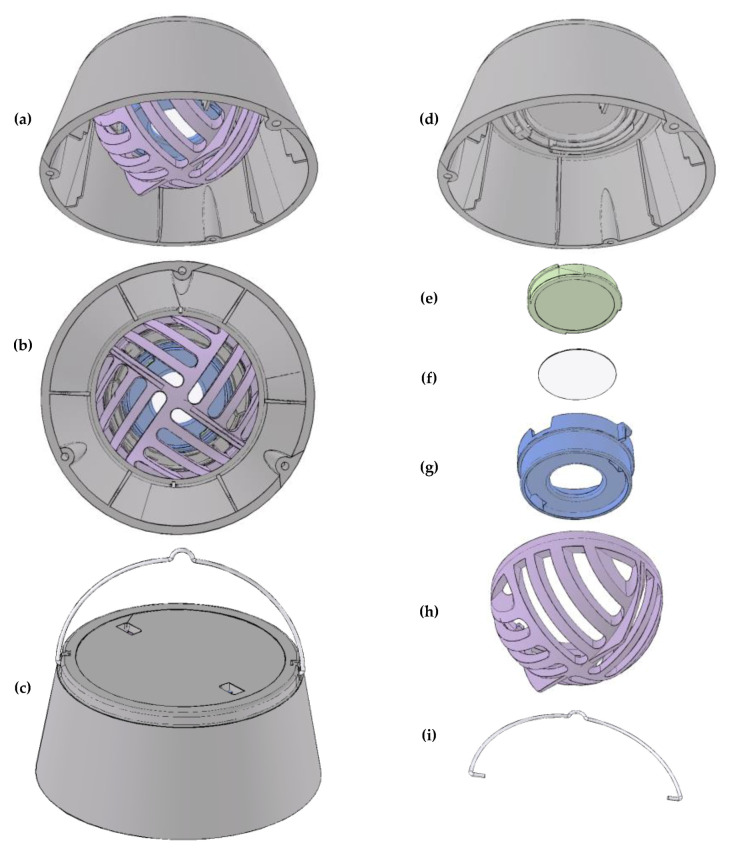
The assembled low-cost passive sampler, shown from (**a**) side, (**b**) bottom, and (**c**) top, and its components, including the (**d**) cap, (**e**) base for paper filter exposure surface, (**f**) paper filter exposure surface, (**g**) clamp for paper filter exposure surface, (**h**) protective cage, and (**i**) wire for hanging sampler.

Figure 1 presents a schematic of the low-cost passive sampler, which consists of a downward-facing paper exposure surface contained and protected by plastic casing.

##### Exposure Surface 

The exposure surface (Figure 1f) is a circle (2.5 cm diameter; 5.1 cm^2^ area) cut from white filter paper (Whatman 1002110 qualitative circle cellulose filter paper, 11 cm diameter, GE Healthcare, Chicago, IL, USA). We selected paper for the exposure surface as a low-cost material with lower glare in lightbox images compared to other materials we tested (i.e., stainless steel, glass, quartz) and high initial reflectance (i.e., white surface without coating). Based on particle size distributions for LAC (e.g., estimated geometric mass mean diameter for BC from biomass burning: ~0.2 μm [39]), we designed the sampler for collection of particles to the exposure surface via diffusion: the exposure surface faces downward to avoid collection of larger particles (e.g., PM with diameter >2.5 μm) via gravitational settling. The filter clamp (Figure 1g) partially covers the paper filter, to provide an internal reference: after removing the exposed paper filter from the sampler, the reflectance of the covered (unexposed to the air) surface area (2.8 cm^2^; 56% of total area) can be directly compared to the reflectance of the uncovered (exposed) surface area (2.3 cm^2^; 44% of total area) from digital images, to estimate the total change in reflectance of the filter due to exposure to air pollution. Additionally, the clamp holds the paper filter exposure surface in a flat position: digital image-based methods for reflectance may be sensitive to curved filter surfaces [22].

##### Casing

The casing consists of four parts: a cap (to protect the exposure surface from turbulence; Figure 1d), a holder for the exposure surface (which has two parts: a base (Figure 1e) and a clamp (Figure 1g)), and a cage (to protect the exposure surface from other contact; Figure 1h). The assembled casing has a “truncated cone” shape with dimensions: diameter of bottom: 8.0 cm, diameter of top: 6.0 cm, height: 3.5 cm, and with mass: 50 g. We selected a truncated cone shape for the sampler casing and a circular shape for the exposure surface to reduce asymmetry in the passive deposition of particles to the exposure surface. To reduce the influence of surface charge on deposition of PM to the exposure surface, we selected a static-dissipative polycarbonate material for the casing. For portability, the holder for the exposure surface is readily removable (by twist-turn) from the cap, and assembled samplers are stackable.

##### Assembly and Deployment

For assembly, the four polycarbonate parts of the casing (Figure 1d,e,g,h) twist and lock into place (without adhesives or other hardware and without tools), and the paper filter exposure surface (Figure 1f) clamps into place between the base (Figure 1e) and clamp (Figure 1g) of the filter holder. Identifying sample labels adhere to the outside of the cap and to the holder for the exposure surface.

For deployment indoors, the sampler can be placed hanging from a hook (from, for example, a ceiling beam) or sitting on a surface (for example, on a table or shelf). For the hanging placement, the sampler hangs by a wire (illustrated in Figure 1c,h) that hooks into the sampler cap. Alternately, for the surface placement, the sampler sits on “stilts” of three screws (length: ~5 cm) threaded in the three openings on the bottom of the sampler cap, to allow flow of air between the sampler cap and the placement surface.

##### Cost Estimate for Sampler

The estimated cost per sampler (Figure 1 hardware) is from ~3 USD (basis: 2000 samplers; injection molding) to ~10 USD (basis: 50 samplers; 3D-printing; Appendix A). That cost estimate includes manufacturing and materials only, and excludes shipping and handling, assembly, and imaging and analyzing the samplers.

#### 2.1.4. Approach for Estimating Sampler Change in Reflectance from Digital Images

Our approach for estimating sampler change in reflectance involves: (1) imaging the sampler exposure surface with a monochrome digital camera in a lightbox, and then (2) analyzing images to estimate the change in “mean pixel intensity” (i.e., change in average grayscale value) of the exposure surface over time.

##### Imaging Sampler Surfaces

To image the sampler surfaces, we used a digital camera as a lower-cost, widely available, easy-to-use sensor covering visible light: LAC strongly absorbs light across the range of visible light wavelengths (~380 nm to ~750 nm) [3]. We used a monochrome (i.e., grayscale or black and white) digital camera because our goal was to measure change in reflectance (i.e., change in grayscale): the monochrome camera provided a higher resolution for grayscale (12-bit pixel intensity or grayscale units: integers from 0 to 4095) compared to color cameras (e.g., for RGB: 8-bit pixel intensity or grayscale units: integers from 0 to 255) used in prior work with active samples [10,22,30,31]. More specifically, we used a monochrome digital camera with 12-bit pixel depth and 14 frames per second shutter speed (Basler acA3800-14um (resolution: 10-megapixel) and Basler puA2500-14um (resolution: 5-megapixel) with a lens (Basler C125-0818-5M (focal length: 8 mm)) with a working distance of 10 cm. We manually adjusted the focus, aperture, and exposure time settings to focus on the exposure surface and to avoid over-saturation. We set the camera to image without gain (to reduce noise introduced per pixel) in monochrome (i.e., grayscale) 12-bit format (to maximize the range of grayscale values per pixel). 

**Figure A2:**
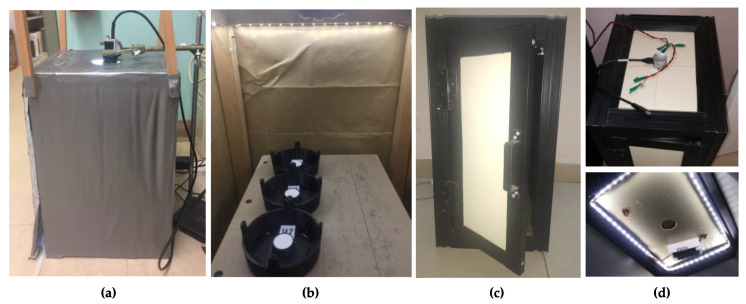
Lightboxes used to image exposure surfaces for the (**a**,**b**) preliminary laboratory testing (in Seattle, WA, USA; lightbox dimensions: 50 cm × 26 cm × 20 cm; structure materials: plywood, cardboard, paper; lighting: flexible LED strip lights (LE 4100057-DW; 24 W, 12 V), camera: digital monochrome 10-megapizel camera (Basler acA3800-14um)) and (**c**,**d**) preliminary field testing (in Hyderabad, India; dimensions: 60 cm × 32 cm × 17 cm; structure materials: wood; lighting: LED strip lights, camera: digital monochrome 5-megapixel camera (Basler puA2500-14um)).

To control image lighting conditions across samples and over time, we constructed a lightbox with low-cost materials, including light-emitting diodes (LEDs) as the source of lighting, a tripod to fix the position of the camera (20 cm above the sample exposure surface), and a black-out shade to further reduce ambient light interference during imaging. We constructed two lightboxes (Figure A2): one used for laboratory testing (in Seattle, WA, USA) and one used for field testing (in Hyderabad, India). As a sensitivity test of lightbox design features (e.g., lighting, dimensions, materials), we used a common set of exposed filters to inter-compare the performance of the two lightboxes (Section 2.2.2).

**Figure A3:**
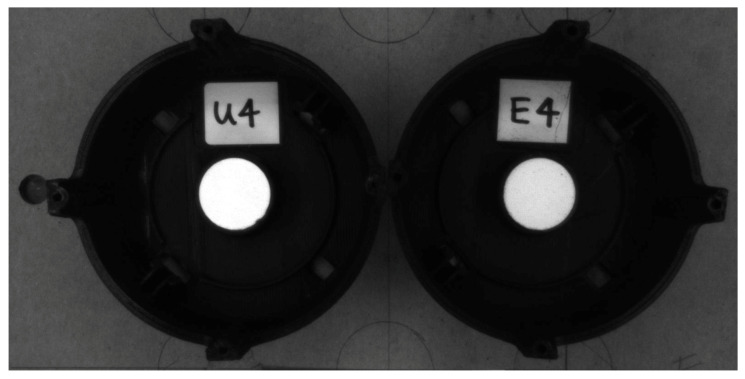
Image layout detail with a reference sampler (on the left; “U4”) and sample (experimental) sampler (on the right; “E4”), side-by-side, inside the lightbox. Samplers shown here, which are the same as in Figure A1c and Figure A2b, differ slightly from the final design; the largest difference is use here of transparent double-sided tape rather than a threaded clamp (Figure 1g) to attach the paper surface to the plastic backing.

To image the low-cost passive sampler exposure surfaces, we placed the exposed sampler with an unexposed reference sampler (stored in a sealed container) in the lightbox, side-by-side, with protective cages (Figure 1h) removed, in fixed positions in the center of the camera field of view (Figure A3). The paper exposure surfaces remained in the casing (i.e., clamped in the exposure surface holder) during imaging, to limit potential contamination of the exposure surface, and to provide an identifying label within the digital image layout. To collect images, we exported 20 frames (format: TIFF).

##### Estimating Change in Sampler Reflectance

Like other optical methods for LAC (e.g., smoke stain reflectometer), the method here estimates the change in reflectance [21]. Our metric for estimating change in reflectance is the change in grayscale value of the exposure surface, measured as pixel intensity (PI; unitless), based on digital images over time. Pixel intensity values in the 12-bit grayscale images are integers ranging from 0 (perfect black; lowest reflectance) to 4095 (perfect white; highest reflectance). To estimate the reflectance of exposure surface at a specific time-point, we calculated the mean pixel intensity of a square area (49-pixel × 49-pixel square; corresponding to approximately 0.4 cm^2^ of the sampler exposure area surface) selected at the center of each exposure surface (passively exposed sample and unexposed reference) for each of the 20 frames. We selected the surface area (49-pixel × 49-pixel square) at the center of the exposure surface to provide an averaging area covering many pixels while avoiding potential edge effects (such as potential contamination from handling the edges of filters, potential shadows from the filter clamp onto the exposure surface in photographs, or other discontinuities in diffusion of particles to the exposure surface near edges). We used 20 frames for calculating mean pixel intensity at each time-point to account for minor fluctuations in lighting conditions among frames. We used MATLAB software (MathWorks, Natick, MA, USA) to calculate the mean pixel intensity of the sample and reference from the digital image files collected at each time-point. We then calculated the absolute change in mean pixel intensity of the passively exposed sample (ΔPI_S[T-0]_) at exposure time T as follows:

ΔPI_S,[T-0]_ = (PI_S,T_ − PI_R,T_) − (PI_S,0_− PI_R,0_), (1)

where PI_S,0_ and PI_R,0_ are the mean pixel intensity of the sample (S) and the reference (R), respectively, before air pollution exposure (exposure time t = 0), and PI_S,T_ and PI_R,T_ are the mean pixel intensity of the sample (S) and the reference (R), respectively, after air pollution exposure (exposure time t = T). Thus, the change in reflectance of each sample is calculated relative to its own baseline reflectance (to account for minor differences in baseline reflectance of filter paper) and to an unexposed reference at each time-point (to account for differences in overall brightness of lighting conditions inside the lightbox over time). The values for our change in reflectance metric (ΔPI; in pixel intensity units [PI] or expressed as a percentage [%] by dividing the value in PI by 4095 PI (i.e., the absolute maximum value)) range from 0 PI (0%; which indicates no change in reflectance) to −4095 (−100%; which indicates a change in reflectance from perfect white to perfect black).

##### Cost Estimate for Approach

The estimated cost of the camera and lightbox for imaging samples (Figure A2c,d hardware) is less than ~500 USD (Appendix A). A single camera and lightbox can be used to image a large number (e.g., thousands) of samples in a central location (e.g., an office or a laboratory) near field locations.

### 2.2. Testing of the Sampler 

Our study primarily aimed to investigate the reproducibility and limits of detection of the low-cost passive method for measuring change in reflectance. Investigating the accuracy of the method was outside the scope of this study. We tested the method for measuring change in reflectance in two conditions: in the laboratory, with shorter term (6 h) exposure to kerosene lamp smoke, and in the field, with longer-term (~8 months) exposure to indoor air pollution in a community that uses solid fuels for cooking. Laboratory testing aimed to investigate the proof-of-concept and the reproducibility of the method with exposure to a single source of LAC air pollution emissions. Field testing aimed to investigate the limits of detection, variability, and reproducibility of the method, as well as practical aspects of suitability of the method for longer-term monitoring indoors. The sampler employed for testing is nearly identical to the final design in Figure 1, with minor differences (Figure A1c, Figure A2b, and Figure A3); the main difference is that for the sampler employed for testing, the paper exposure surface was held to the cap using transparent double-sided tape (Figure A1c), rather than via a threaded cap (Figure 1g).

#### 2.2.1. Laboratory Testing

**Figure A4:**
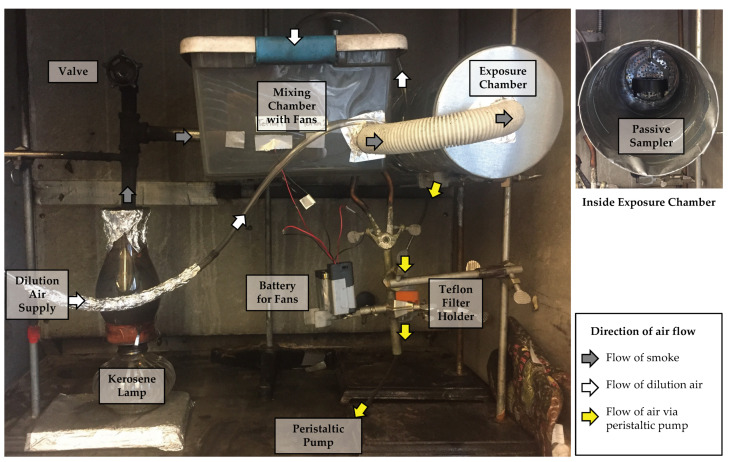
Design of exposure chamber with kerosene lamp as source of black carbon air pollution.

We tested the change in reflectance of low-cost passive samplers with exposure to LAC air pollution in the laboratory (Figure A4). We used a kerosene (paraffin) hurricane lamp with a cloth wick as the source of LAC for several reasons: kerosene lamps are low-cost and convenient to use; kerosene lamps are a common source of air pollution in low-income households without access to electricity (an estimated 500 million households used kerosene lamps for lighting in 2005 [40]); and, LAC (more specifically, BC) is the major component of PM emitted by kerosene lamps (e.g., >88% of PM mass is BC [41]). We operated the kerosene lamp inefficiently (i.e., with the cloth wick extended ~3 mm beyond the wick-guide) to increase the LAC emission rate. To stabilize the air pollution concentration during exposure testing, we used filtered dilution air (flow rate: 5.8 L min^−1^) and three battery-operated fans in a mixing chamber (volume: 0.06 m^3^) prior to the exposure chamber (volume: 0.05 m^3^). To estimate the particulate matter (PM) concentration in the exposure chamber, we collected gravimetric samples using PTFE filters (Pall Laboratory R2PJ037, air sampling PTFE membrane, 37 mm diameter, Pall Corporation, Port Washington, NY, USA) and a peristaltic pump (SidePak 530, TSI Incorporated, Shoreview, MN, USA; flow rate: 0.9 L min^−1^). The PTFE filters were pre-conditioned in a temperature- and relative humidity- controlled environment for at least 2 weeks prior to pre-weighing (i.e., weighing before sample collection) and for at least 24 h prior to post-weighing (i.e., after sample collection). Filters were handled with non-serrated plastic forceps and weighed using a gravimetric balance (UMT-2, Mettler-Toledo, Greifensee, Switzerland) with accuracy of +/−0.5 μg.

During exposure testing, we placed three samplers in the exposure chamber, hanging from hooks placed at the top of the exposure chamber (Figure A4). We imaged the three low-cost passive samplers in the lightbox (Figure A2a,b) at baseline, and at 20-min exposure intervals, repeating for a total of 360 min of exposure. We selected 20-min exposure intervals based on practical requirements for maintaining consistent conditions in the exposure chamber between sampling time-points (e.g., maintaining constant wick height for the lamp, avoiding excessive accumulation of soot in the kerosene lamp and connective tubing during exposure periods, allowing time for smoke to fill the chamber between sampling time-points). Between each 20-min exposure testing interval, we stored the samplers in sealed containers.

We evaluated proof-of-concept by investigating whether the passive low-cost samplers experienced a measurable change in reflectance in proportion with exposure time in the kerosene smoke exposure chamber. We analyzed the reproducibility of the low-cost passive approach by calculating the correlation (Pearson’s coefficient, *r*; Spearman’s rank coefficient, *s*) and root mean square error (RMSE) among the triplicate samplers’ estimated change in reflectance at each time-point.

#### 2.2.2. Field Testing

**Figure A5:**
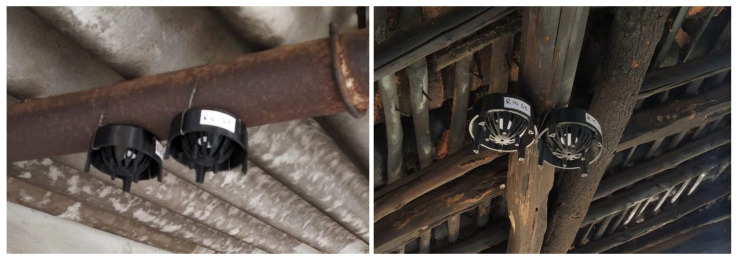
Deployment of paired low-cost passive samplers side-by-side in two indoor locations hanging from (**left**) a metal ceiling and (**right**) a wood ceiling.

We tested the change in reflectance of samplers with exposure to air pollution over ~8 months in 20 indoor locations in households near Hyderabad, India, in a community that uses solid fuels for cooking. In each monitoring location (*n* = 20), we placed two samplers together, side-by-side, hanging (typically from a ceiling beam), away (distance: >1 m) from air pollution emission sources such as stoves and generators and from sources of higher air flow (such as fans) (Figure A5). We imaged the samplers at baseline (day 0) and at approximately monthly intervals (after the following cumulative days of exposure: 33, 55, 90, 118, 173, 209, 258) during the study period (30 December 2018 to 14 September 2019). At each sampling time-point, we collected the samplers from the 20 field locations for imaging in the lightbox (Figure A2c,d) in an off-site office. We stored the samplers in sealed plastic bags during transport and before and after imaging. To investigate saturation behavior of the sampler exposure surfaces, we placed an additional (i.e., third) sampler at one monitoring location (selected based on observed largest change in surface reflectance) after 118 days of exposure until the end of the study period. After the study period, as a sensitivity test of lightbox performance, we imaged the samplers in the lightbox used for laboratory testing (in Seattle, WA, USA; Figure A2a,b).

We investigated the lower and upper limits of detection by tracking the change in mean reflectance of samplers over time. We evaluated the reproducibility of the low-cost passive sampler approach by calculating the correlation (Pearson’s coefficient, *r*; Spearman’s rank coefficient, *s*), coefficient of variation (CV) and root mean square error (RMSE) between paired samplers. We analyzed variability among samplers (by comparing change in mean reflectance for each pair of samplers), variability among locations (by comparing change in mean reflectance across the 20 locations), and variability over time (by comparing change in mean reflectance over sampling time-points). We compared reflectance results from the two lightboxes (Figure A2a,b in Seattle, WA, USA; Figure A2c,d in Hyderabad, India) at the end of the study period (258 days of exposure) to investigate sensitivity of results to imaging method.

## 3. Results

### 3.1. Laboratory Testing

**Figure 2:**
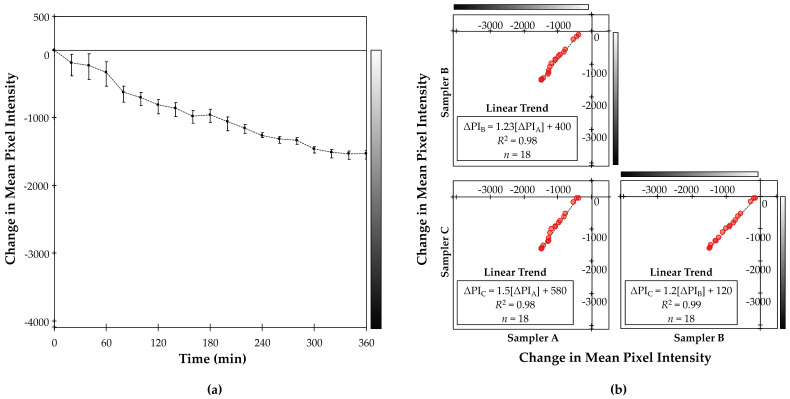
Change in reflectance of three samplers with exposure to kerosene smoke in a laboratory chamber. Change in reflectance (measured by change in mean pixel intensity (ΔPI; unitless) and illustrated by grayscale) ranges from 0 (indicating no change in grayscale) to −4095 (indicating a change in from perfect white to perfect black). (**a**) Average change in reflectance of samplers versus time in kerosene smoke exposure chamber. Points indicate the mean and error bars indicate the range of change in reflectance for the three samplers placed together in the exposure chamber. (**b**) Scatterplot matrix of change in reflectance of three samplers (Samplers A, B, and C) placed together in the exposure chamber.

**Figure A6:**
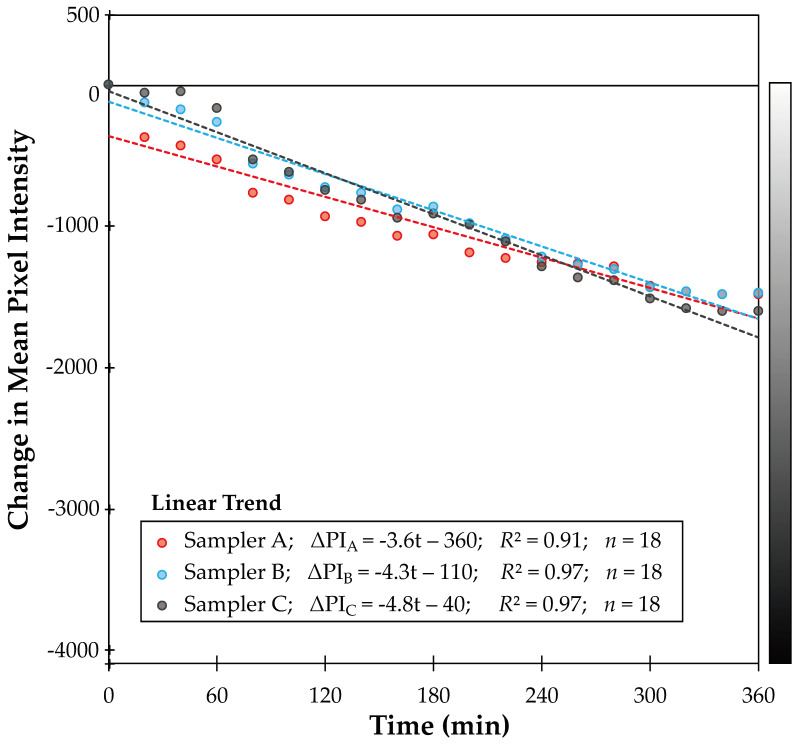
Linear regression models predicting changes in reflectance for three samplers (A, B, C) based on exposure time in the kerosene smoke chamber in the laboratory. Change in reflectance (measured by change in mean pixel intensity (ΔPI; unitless) and illustrated by grayscale) ranges from 0 (indicating no change in grayscale) to −4095 (indicating a change in grayscale from perfect white to perfect black). Points indicate estimated change in reflectance for each sampler for each sampling time-point (18 sampling time-points).

Low-cost passive samplers generally decreased in reflectance (i.e., “darkened”) in proportion with exposure time in the kerosene smoke chamber (Figure 2). Based on analysis of the gravimetric filter samples, the kerosene smoke exposure chamber experienced very high PM concentrations (average: 540 mg m^−3^; i.e., ~10,000× the World Health Organization (WHO) 24-h guideline for PM_2.5_ (25 μg m^−3^ [42])). Samplers’ rates of change in reflectance were relatively consistent over time: the mean rate of change (three samplers, each with 18 time-points) was −4.2 PI min^−1^ (−0.10% min^−1^) (interquartile range: −1.6 PI min^−1^ (0.038% min^−1^) to −6.2 PI min^−1^ (−0.15% min^−1^); CV: 99%). For the three low-cost passive samplers over 17 changes-over-time, 42% of the time, the rate of change in reflectance was within 2 PI min^−1^ of the overall mean rate of change in reflectance (−4.2 PI min^−1^). Linear regression models predicting change in reflectance based on exposure time for each sampler demonstrated high goodness-of-fit (*R^2^* range: 0.91 to 0.97; Figure A6). This pattern (Figure 2a) supports preliminary proof-of-concept: the low-cost passive samplers experienced a measurable and relatively consistent change in reflectance with exposure to high concentrations of a single source of LAC.

Triplicate low-cost passive samplers closely agreed: changes in reflectance were highly correlated among the triplicate samplers (Figure 2b; Pearson’s correlation coefficients (*r*) > 0.99; Spearman’s rank correlation coefficients (*s*) > 0.99; Table A3) with moderate variability (mean CV: 32%; interquartile range in CV: 16% to 39%) and error (mean RMSE, calculated relative to mean of triplicate samplers: 86 PI (RMSE/mean: 8.6%); Table A4) among the triplicate samplers. Variability in change in reflectance (calculated using Equation (1)) among triplicate samplers was generally lower for longer versus shorter exposure times (Figure 2a). For example, the mean CV for the first three sampling time-points was 70% versus 23% for the last three sampling time-points. This pattern of close agreement among triplicate low-cost passive samplers suggests excellent reproducibility of the low-cost passive sampler approach for measuring change in reflectance, particularly for higher levels of exposure to LAC.

### 3.2. Field Testing

**Figure 3:**
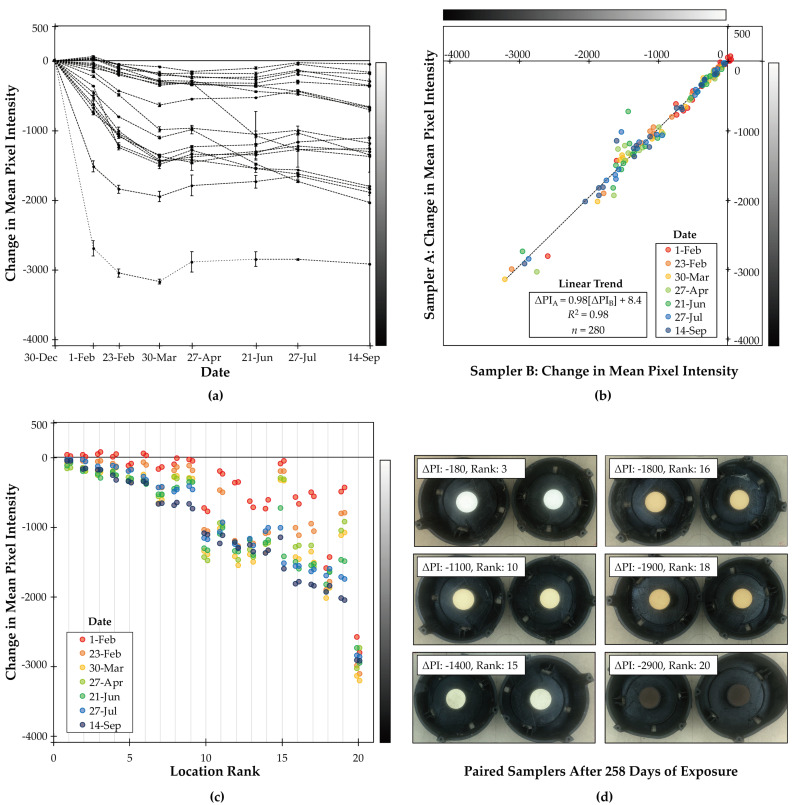
Change in reflectance of paired samplers in 20 indoor locations during 258 days in a community that uses solid fuels. Change in reflectance (measured by change in mean pixel intensity (ΔPI; unitless) and illustrated by grayscale) ranges from 0 (indicating no change in grayscale) to −4095 (indicating a change from perfect white to perfect black). (**a**) Average change in reflectance of samplers at each location (*n* = 20) versus time (7 sampling dates). Points represent the average and error bars indicate the range of darkening for the two samplers placed together at each location. Each line represents one location. (**b**) Scatterplot of change in reflectance of samplers placed at the same location (Sampler A vs Sampler B) by sampling date (*n* = 280). (**c**) Variability in change in reflectance among locations and within locations by sampling date. Locations are plotted in rank order (smallest to largest change in reflectance, 1 to 20) based on average change in reflectance of the paired samplers on 14 September (day 258 of exposure). Points represent each sampler at each location. (**d**) Paired samplers (after 258 days of exposure) with total change in mean pixel intensity (average of paired samplers) and rank (smallest to largest change in reflectance, 1 to 20) to illustrate range in change of exposure surface reflectance across six locations.

Overall, low-cost passive samplers decreased in reflectance during the ~8-month study period in 20 indoor locations (Figure 3a). All low-cost passive samplers (*n* = 40) decreased in reflectance by at least 1% (−41 PI) after ~2 months (baseline to 55 days) of exposure. After ~8 months of exposure (baseline to 258 days), 70% (*n* = 28) of samplers decreased in reflectance by at least 10% (−410 PI) and 55% (*n* = 22) decreased by at least 25% (−1020 PI). 

Although all samplers decreased in reflectance between baseline and the end of the study period (median change: −1120 PI (−27%); interquartile range: −360 PI (−8.8%) to −1800 PI (−44%)), some samplers increased in reflectance between specific sampling time-points, to a comparatively minor extent (relative to the overall trend of decreased reflectance). For example, during the first sampling period (baseline to day 33), 25% (*n* = 10) of samplers increased in reflectance, but the median increase in reflectance among those samplers (+33 PI (+0.82%)) was relatively small (7.7%) compared to the median decrease among the remaining samplers (*n* = 30; −440 PI (−11%)). That result is consistent with the measurement approach having some degree of uncertainty in estimated changes in reflectance (e.g., potentially due to inconsistencies in lighting or in positions of samples within the lightbox between imaging time-points). When air pollution exposure is comparatively low, the estimated change in reflectance is small, and may be small enough that, when combined with measurement uncertainty, yields a result suggesting a minor increase in reflectance. 

The rate of change in reflectance (i.e., rate of darkening) generally slowed during the study period (Figure 3a). The median rate of change in reflectance for the entire study period was −4.4 PI day^−1^ (interquartile range: −1.4 PI day^−1^ to −7.0 PI day^−1^), but that rate was generally larger during the first months of exposure (December 2018 through March 2019; approximate winter season) and smaller during the last months of exposure (April 2019 through September 2019; approximate spring and summer seasons): the median rate of change in reflectance declined from −5.7 PI day^−1^ (interquartile range: −0.37 day^−1^ to −19 PI day^−1^) during the first sampling period (baseline to day 33; approximate month of January) to –3.2 PI day^−1^ (interquartile range: −1.3 day^−1^ to −4.7 PI day^−1^) during the last sampling period (day 209 to 258; approximate month of August). 

The slower rate of darkening (i.e., the decline in rate of change in reflectance) during summer months could be attributable to multiple potential factors, including (1) lower air pollution concentrations during the summer months, and/or (2) saturation of sampler exposure surfaces. Consistent with the first hypothesis, local air pollution emissions in this region typically decline in warmer months, as residential use of solid fuels for heating declines [43]. Consistent with the second hypothesis, the following preliminary testing at one indoor location suggests that samplers may have saturated during the first four months of the study period. A third unexposed sampler placed at the indoor study location with the largest change in reflectance at the beginning of the fifth sampling period (day 118) experienced a change in reflectance of –2100 PI (–52%) by the end of the fifth sampling period (i.e., during day 118 to day 173), whereas the original paired samplers at that location (continuously exposed since baseline; mean change in reflectance at day 118: –2900 (–71%)) experienced an average change in reflectance of +40 PI (1.0%) during the same time period. The new unexposed sampler experienced a much larger (~70 times larger) change in reflectance relative to the previously exposed samplers during the same time-period; that outcome is consistent with saturation of the exposure surface before four months of exposure and/or before experiencing a change in reflectance of –2900 PI (–71%).

Based on these patterns of changes in reflectance over time (Figure 3a), for this sample of indoor locations in this community that uses solid fuels, the minimum time-scale for monitoring using the low-cost passive sampler (indicating the lower limit of detection) appears to be less than two months (given that all samplers experienced a measurable change in reflectance (at least a 1% change in grayscale) after 55 days of exposure), and the maximum time-scale for monitoring (indicating the upper limit of detection) appears to be less than four months (given that preliminary testing at one location indicated saturation effects by day 118 of exposure). Thus, initial testing suggests that the low-cost passive sampler method could be appropriate for longer-term low-burden monitoring: low-cost passive samplers may be left in place for several weeks before replacing exposure surfaces (to avoid potential saturation effects).

The indoor locations varied substantially in reflectance at each time-point (Figure 3c; Table 1). After the first sampling period (baseline to day 33), the range in change in reflectance (mean based on paired samplers) across locations (*n* = 20) was 2800 PI (-2700 PI to +60 PI; CV: 150%), and, after the last sampling period, was 2900 PI (–2900 PI to –52 PI; CV: 71%). Variability in mean reflectance among locations at the same time-point (mean CV: 93%) was larger than variability in reflectance for the same location over time (mean CV: 45%) and larger than variability for repeated measurements at the same location at the same time-point (mean CV: 11%). This pattern of substantial variability in reflectance among locations (relative to variability over time and between repeated measurements) suggests ability of the low-cost passive method to differentiate between locations with higher versus lower longer-term average air pollution exposures (as estimated here as higher versus lower changes in reflectance).

In addition to variability in quantitative measures of reflectance (based on grayscale digital images), locations displayed high variability in qualitative color of paired samplers (Figure 3d). The present study used only monochrome images to quantify differences in grayscale among filters; future research could potentially explore differences in color among filters, an approach applied in prior work [10,22,30].

Paired samplers closely agreed (Figure 3b). Estimated changes in reflectance were highly correlated for paired samplers at the same location (*r* = 0.99; *s* = 0.99; Table 2). Differences in estimates between paired samplers at the same location were generally modest compared to the range among locations (Table 1) and compared to mean reflectance (for all samplers and sampling dates: RMSE: 110 PI; RMSE/mean: 8.8%; Table 2). This general pattern of close agreement between paired samplers suggests the ability of the low-cost passive sampler approach to provide reproducible (i.e., high-precision) estimates of reflectance.

Testing explored potential practical challenges for long-term placement indoors. During the ~8-month exposure period, all low-cost passive samplers remained intact (i.e., assembled with the paper exposure surface in place), suggesting that samplers were appropriately durable for long-term placement indoors. Similarly, all samplers remained installed (i.e., no samplers were lost during the 8-month testing period), suggesting that samplers were appropriately unobtrusive when placed hanging from a ceiling indoors. However, for this type of long-term placement, samplers may be contaminated by insects or spiders over time: at the end of the study period, we observed spiders’ webs on the cages of two samplers (5% of samplers). Thus, samplers may be more suitable for use during seasons when and in geographic regions where insect and spider activity is low.

**Figure A7:**
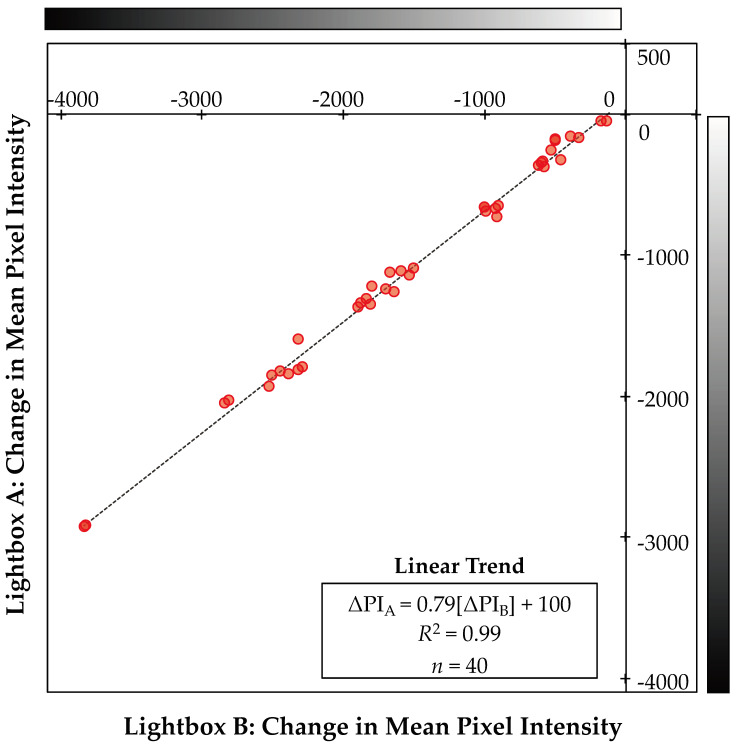
Correlation of change in reflectance of samplers (*n* = 40) after 258 days of exposure in 20 indoor field locations, as estimated using two different lightboxes: lightbox used for field testing (Lightbox A in Hyderabad, India; Figure A2c,d) and lightbox used for laboratory testing (Lightbox B in Seattle, WA, USA; Figure A2a,b). Change in reflectance (measured by change in mean pixel intensity (PI; unitless) and illustrated by grayscale) ranges from 0 (indicating no change in grayscale) to −4095 (indicating a change from perfect white to perfect black).

As a sensitivity test on the method for estimating reflectance, we compared estimates of total change in mean reflectance for the low-cost passive samplers (*n* = 40; after the ~8-month exposure period) based on two lightboxes. One light box was located in Hyderabad, India; the other in Seattle, United States. The two light boxes differed in dimensions, materials, lighting source, and camera model (Figure A2). Nevertheless, agreement of results from the two lightboxes was excellent (Figure A7). Close agreement between the two sets of estimates (*R^2^* = 0.99; RMSE/mean: 18%) suggests that estimated change in reflectance using this low-cost passive method is not highly sensitive to the lightbox design or camera selection.

## 4. Discussion

### 4.1. Performance, Cost-Effectiveness, and Ease-of-Use 

#### 4.1.1. Performance

During shorter-term laboratory testing with high concentrations of a single source of LAC and during longer-term field testing with environmental concentrations of multiple sources of PM (in indoor locations near Hyderabad, India), the low-cost passive samplers experienced measurable (|ΔPI| > 1% (41 PI)) and reproducible (repeated samplers: *r* = 0.99), changes in reflectance with exposure time. Based on preliminary analysis of limits of detection in 20 indoor locations in a community that uses solid fuels, the timescale for each round of sampling (i.e., before filter replacement) appears to be ~1 to ~3 months, which reflects longer-term average conditions. For multiple months of sampling, samplers were appropriately durable and, to our knowledge, unobtrusive when placed indoors hanging from ceilings. Here, preliminary testing focused on reproducibility, limits of detection, and general suitability for longer-term monitoring indoors; further testing (discussed in Section 4.2) is needed to assess other aspects of performance, such as the accuracy of the method.

#### 4.1.2. Cost-Effectiveness

The method here, which combines a low-cost sample collection method (passive) with a low-cost analysis method (digital image-based), is cost-effective compared to other available methods for monitoring LAC in terms of specialized equipment requirements (for sample collection and for analysis) and in terms of filter replacements over a long-term sampling period (Appendix A). The equipment cost for sample collection (~10 USD per sampler) is an order of magnitude less than other available low-cost real-time LAC sensors (e.g., ~500 USD [11]) and multiple orders of magnitude less than established real-time LAC sensors (e.g., ~7000 USD for a micro-aethalometer). Like other filter-based and digital image-based methods (e.g., [10]), the cost for the analysis equipment (digital camera with lightbox: ~500 USD) is comparable to or less than other available time-integrated average filter-based optical methods for LAC (e.g., ~4000 USD for a smoke stain reflectometer, ~8000 USD for optical transmissometer). As applied here, the cost for filters for the passive method is substantially lower compared to active filter-based optical or thermal-optical methods over longer-time sampling periods: for one sampling location, filters for the passive sampling method would cost <1 USD per location per month (assuming one qualitative paper filter per month (0.03 USD per filter)) versus ~60 –300 USD per location per month (assuming 30 daily 24-h samples collected on either all quartz (~2 USD per filter) or all PTFE filters (~10 USD per filter)) for active sampling methods. 

#### 4.1.3. Ease-of-Use

For sampling using the fully passive method, the time required over a period of weeks to months is minimal: no maintenance (such as battery or filter replacement) is needed, and the samplers can be left in place for weeks to months at a time. For placement indoors, the fully passive samplers are unobtrusive in that they are compact (dimensions: <10 cm) and produce no noise. Additionally, privacy concerns related to using these fully passive long-term samplers indoors (e.g., in homes, schools, workplaces) may be less than for active real-time methods in that they operate fully offline (i.e., are not connected to the Internet) and record only a long-term time-integrated average estimate of LAC levels (i.e., do not record data that could be used to infer time-activity patterns [15]).

For analysis, imaging samples with a camera requires minimal time (~15 samples per hour can be imaged using one lightbox) and training (digital cameras are widespread technology), and can be done in any indoor environment with power (i.e., imaging does not require specialized laboratory facilities; imaging can be at a central indoor location (office, classroom, etc.) for field campaigns). Additionally, using a digital camera for imaging can reduce risk of contamination compared to other optical filter-based methods for LAC (such as smoke stain reflectometer or colorimeter): imaging with a camera requires less direct handling of samples (i.e., exposure surfaces remain clamped into passive sampler during imaging; they are not removed and transferred as for other methods) and requires no direct contact with sample surfaces [22]. 

### 4.2. Limitations, Further Testing Needs, and Potential Improvements

#### 4.2.1. Limitations

Like other passive methods for air sample collection, the method here is limited in that sample collection may be sensitive to surrounding air flow rates, and in that the time-scale needed for sampling is dependent on average air pollution concentrations. First, because air flow rates influence the rate of LAC deposition to the passive exposure surface [26,44], the samplers may be limited to placement indoors, away from sources of variable air flow (ventilation systems, fans, etc.). Second, because the ambient LAC concentration influences the rate of LAC deposition to the passive exposure surface, the time-scale for sampling is dependent on expected long-term average LAC concentrations. In locations with very low LAC concentrations, the time-scale to observe a measurable change in reflectance using this passive method may be impractically long (~years), whereas in very high LAC concentration environments, the sampler surface may quickly (~days, ~week) reach saturation.

Like other time-integrated average filter- and digital image-based methods for LAC, the method here is limited in that filter reflectance is used as an indirect measure for LAC levels and in that the estimate of reflectance represents long-term, time-integrated average conditions. Additionally, like other reflectance-based methods, the method assumes that all change in filter reflectance is due to LAC exposure. 

#### 4.2.2. Further Testing Needs 

Further testing is needed to determine: (A) accuracy of method; (B) calibration requirements; (C) suitability of method for community science applications. First, although initial testing indicated that the low-cost passive method is precise (in that repeated measurements strongly agree), the accuracy of the method has not been investigated. Further testing can investigate accuracy by comparing reflectance estimates from the passive digital image-based method with existing optical filter-based methods for reflectance (such as smoke-stain reflectometer) and with active real-time methods for LAC (such as an aethalometer). Further testing of accuracy can also investigate potential sensitivity of paper exposure surfaces to other environmental conditions (e.g., light, heat, relative humidity) during long-term monitoring indoors and further investigate the saturation behavior of filter surfaces. Second, as part of such comparisons with alternate methods for LAC, further testing is needed to determine appropriate methods for calibration, so that the estimates of reflectance from the low-cost passive method can be reliably related to time-integrated average LAC levels. LAC estimates using image-based methods for reflectance may be sensitive to the site, season, pollution source (e.g., fuel type), and/or reference method selected for calibration [36]. Third, further testing is needed to explore the suitability of the low-cost passive sampler for large-scale monitoring via citizen science campaigns and community-led monitoring [45,46,47]. Here, the research team assembled, deployed, imaged, and analyzed samplers: further testing can explore potential suitability of the method for use by citizen scientists.

#### 4.2.3. Potential Improvements

Future designs could increase portability (e.g., by using a folding, battery-powered lightbox, and/or cellphone camera [10,30]) and automation (e.g., using advanced computing methods or smartphone applications [30]) of image collection and analysis. Additionally, future designs could potentially combine the passive sampling approach here with other analysis methods. As one example, future research could explore using the passive sampling approach with chemical analysis methods (which may require a different material for the exposure surface (e.g., quartz or PTFE filter) rather than the paper filter used here for optical analysis). As a second example, the optical analysis here could be extended to incorporate color metrics (e.g., using RGB [10,22,30], HSV [33], or CIE-Lab [32] color models) in addition to (or as alternatives to) the grayscale metrics (i.e., pixel intensity) for reflectance used here. Specifically, future work using color-based metrics could test whether this method can be used to reliably differentiate other components of PM_2.5_ (e.g., BrC versus BC) and, in turn, to differentiate potential sources of exposure (e.g., air pollution exposure from burning biomass versus fossil fuels [48]).

### 4.3. Potential Applications of Method

The method here is intended as a low-cost, easy-to-use supplement or complement (rather than as a direct replacement) for existing monitoring methods for LAC, extending the number of possible monitoring locations and time-periods to enable large-scale, long-term monitoring indoors. Potential applications for this method include large-scale cohort studies, intervention studies, or community science campaigns, for which a long-term (~months) time-integrated quantitative measure of relative LAC levels is desired for a large number (e.g., thousands) of locations, including locations without access to reliable electricity. At present there are no technical options available for that type of long-term monitoring at that scale.

Because the method relies on passive exposure, we expect that potential applications are limited to indoor environments (where air flow rates would be relatively consistent compared to outdoor locations) with high expected LAC concentrations, such as in households that rely on solid fuels for cooking and/or heating. The range of concentrations experienced in those environments is wide (often covering at least an order of magnitude). As examples, based on available data, among 29 kitchens in rural Ghana, 24-h average BC concentrations ranged from 2.8 to 29 μg m^−3^ [49]; among 44 living rooms (lit with hurricane kerosene lamps) in rural Uganda, 24-h average BC concentrations ranged from 0.08 to 22 μg m^−3^ [50]; and, among 17 kitchens in rural India, hourly average BC concentrations ranged from 5.4 to 35 μg m^−3^ [51]. For understanding patterns across a wide range of air pollution concentrations, even a crude measure of indoor LAC levels might be useful.

## 5. Conclusions

Toward developing a low-cost easy-to-use long-term monitoring method for household air pollution, we designed and tested a passive sampler for indoor LAC levels. Building on prior work, this design combines a low-cost sample collection approach (via passive exposure) with a low-cost analysis approach (via digital images) to estimate the change in reflectance of a passively exposed surface. The passive sampler is low-cost (e.g., cost for materials less than ~10 USD per sampler, which is an order of magnitude lower compared to other available lower-cost LAC sensors and 2–3 orders of magnitude lower compared to standard thermal-optical sensors and optical real-time LAC sensors), easy-to-use (compact, light-weight, fully passive, easy to assemble and to deploy), and suitable for longer-term (~1–3 months) monitoring in households that use solid fuels. The initial testing described here suggests that the low-cost passive sampler estimates changes in reflectance with high precision. Further testing is needed to investigate the accuracy compared to other methods for estimating reflectance.

## Figures and Tables

**Table 1:** Variability in change in reflectance (change in mean pixel intensity (ΔPI; unitless)) by sampling date among indoor field locations (*n* = 20; based on mean of paired samplers at each location) during 258 days in a community that uses solid fuels.

	Variability within Locations		Variability among Locations	
Exposure Time (Sampling Date)	Mean absolute difference ^a^ in ΔPI between paired samplers at same location (range of absolute differences) [PI]	Mean CV ^b^ [%]	Absolute difference ^c^ in ΔPI among location averages (range of location averages) [PI]	CV ^b^ [%]
33 days (1 February)	63 (5.0 to 220)	34	2800 (−2700 to +60)	150
55 days (23 February)	45 (1.0 to 120)	8.2	3000 (−3000 to −46)	110
90 days (30 March)	58 (7.0 to 160)	6.5	3100 (−3200 to −83)	85
118 days (27 April)	92 (14 to 310)	8.2	2700 (−2900 to −150)	81
173 days (21 June)	96 (4.0 to 700)	8.7	2700 (−2900 to −100)	73
209 days (27 July)	63 (1.0 to 510)	8.3	2800 (−2900 to −32)	80
258 days (14 September)	61 (5.0 to 450)	5.2	2900 (−2900 to −52)	71

^a^ Mean and range (minimum to maximum) in absolute differences in change in mean pixel intensity (PI) between the two paired samplers placed together at each location, among the 20 locations; ^b^ Coefficient of variation (absolute value); ^c^ Absolute difference and range (minimum to maximum) in average change in mean pixel intensity (PI) (of the two paired samplers at each location) among the 20 locations.

**Table 2:** Correlation and root mean square error (RMSE ^a^) in change in reflectance (change in mean pixel intensity (ΔPI)) between paired samplers in 20 indoor locations, by sampling date, and by quintile of change in reflectance (ΔPI).

			Correlation in ΔPI		Error in ΔPI	
Samplers	Number of pairs, *n*	MeanΔPI [PI]	Pearson’s coeff., *r*	Spearman’s rank coeff., *s*	RMSE ^a^ [PI]	RMSE ^a^/mean [|%|]
*All samplers, all sampling dates*	140	−850	0.99	0.99	110	8.8
*Samplers by sampling date:*						
1 February (range: −2700 to +60)	20	−440	0.99	0.99	41	9.3
23 February (range: −3000 to −46)	20	−720	0.99	0.99	30	4.2
30 March (range: −3200 to −83)	20	−930	0.99	0.99	37	4.0
27 April (range: −2900 to −150)	20	−890	0.98	0.98	140	1.6
21 June (range: −2900 to −100)	20	−970	0.97	0.97	90	9.3
27 July (range: −2900 to −32)	20	−930	0.99	0.98	62	6.7
14 September (range: −2900 to −52)	20	−1100	0.99	0.98	57	5.3
*Samplers by quintile ΔPI:*						
Q1 (range: +60 to −180)	28	−83	0.89	0.85	18	22
Q2 (range: −180 to −360)	28	−280	0.87	0.85	20	7.1
Q3 (range: −360 to −1100)	28	−670	0.96	0.92	33	4.7
Q4 (range: −1100 to −1400)	28	−1200	0.15	0.20	110	8.5
Q5 (range: −1400 to −3200)	28	−2000	0.98	0.98	220	11

^a^ RMSE calculated relative to the mean of the paired samplers’ change in reflectance.

## Appendix A. Supporting Information for Design of Sampler and Approach

Table A1 presents cost estimates for the low-cost passive sampler. In total, the estimated cost for materials per sampler (including polycarbonate casing, one stainless steel wire hook for installation, one exposure surface (2.5 cm diameter circle cut from 11 cm diameter qualitative filter), and labels (5 cm length of 6 mm water resistant label tape per sampler)) ranges from ~3 USD (assuming injection molding a batch of 2000 units) to ~10 USD (assuming 3D-printing a batch of 50 units). This cost estimate reflects only the materials for samplers and does not include the equipment to image and analyze the samplers (camera, computer, software, lightbox; discussed next) and does not include the labor to deploy samplers, to image samplers, and to analyze data.

**Table A1:** Cost estimates for the low-cost passive sampler.

Component	Manufacturer and Product	Approximate Cost per Unit [USD]
*Sampler hardware* ^1^		
3-D printed		
Batch of 50	Custom	9.6
Injection molded		
Batch of 1000	Custom	3.2
Batch of 2000	Custom	2.5
Batch of 5000	Custom	2.0
*Exposure surface*	Whatman 1002110	0.03
*Labels*	Brother TZE211	0.06

^1^ Sampler hardware includes the polycarbonate casing and the wire hook for installation. The polycarbonate casing cost depends on manufacturing method and batch size.

Table A2 presents estimated fixed costs (for specialized materials only) for imaging and analyzing the change in reflectance of the low-cost passive sampler, based on the set-up used in the field testing results here (Figure A2c,d). In total, the estimated cost for imaging and analyzing the low-cost passive sampler is ~440 USD. This cost estimate reflects only the specialized materials for the imaging and analysis methods and does not include the general computing resources (computer, software, data storage) or the labor to deploy samplers, to image samplers, and to analyze data.

**Table A2:** Cost estimates for equipment for analyzing the low-cost passive sampler.

Component	Manufacturer and Product	Approximate Cost per Unit [USD]
*Digital camera*	Basler puA2500-14um	320
*Lightbox*	Custom	120

## Appendix D. Supporting Information for Laboratory Testing Results

**Table A3:** Correlation matrix for change in reflectance (change in mean pixel intensity (ΔPI; unitless)) by kerosene smoke chamber exposure time-point (*n* = 18) among triplicate low-cost passive samplers (*n* = 3; Sampler A, Sampler B, Sampler C). Lower panel lists the Pearson’s correlation coefficients (*r*), and the upper panel lists the Spearman’s rank coefficient (*s*).

	Sampler A	Sampler B	Sampler C
**Sampler A**	-	1.000	0.998
**Sampler B**	0.992	-	0.998
**Sampler C**	0.992	0.999	-

**Table A4:** Root mean square error (RMSE), calculated relative to the mean value (among triplicate low-cost passive samplers), in estimated change in reflectance (change in mean pixel intensity (ΔPI; unitless)) for all sampling time-points (*n* = 18).

	Sampler A	Sampler B	Sampler C	Mean
Root mean square error (RMSE) [PI]	120	56	83	86
Mean [PI]	−1100	−930	−960	−990
RMSE/Mean [%, absolute value]	11	6.0	8.6	8.6
